# Development and Application of Technology for Neural Circuit Visualization - Secondary Publication

**DOI:** 10.31662/jmaj.2024-0019

**Published:** 2024-04-05

**Authors:** Shigeo Okabe

**Affiliations:** 1 Department of Cellular Neurobiology, Graduate School of Medicine, The University of Tokyo, Tokyo, Japan

**Keywords:** Neuron, Cytoskeleton, Synapse, GFP, Live imaging

## Abstract

The dynamics of neurite extension and synaptic connections are central issues in neural circuit research. The development of technologies for labeling purified cytoskeletal proteins with fluorescent dyes and introducing them into living neurons using microinjection greatly facilitated our understanding of cytoskeletal dynamics in neuronal axons. Imaging data showed that the cytoskeleton repeatedly polymerized and depolymerized within the axon, and elongation was driven by the new cytoskeleton formed at the axon tip. This finding significantly revised previously proposed models that explained slow axonal transport.

After the discovery of green fluorescent protein (GFP), its potential application to the live imaging of neurons was recognized in the 1990s, and a new method for visualizing synapses using GFP-tagged postsynaptic scaffolding molecules was established. This method revealed the continuous turnover of synapses during development, which overturned the established theory that synapses are highly stable once they are formed. Live imaging of synapses also demonstrated that the molecular composition of synapses changes rapidly, driven by the rapid replacement of synaptic molecules. Fluorescence measurement of single GFP molecules enabled estimation of the absolute number of postsynaptic molecules in a single synapse. Furthermore, in multiple mouse models of autism spectrum disorders (ASDs), enhanced synapse turnover was detected as a common circuit-level phenotype. This study provides solid experimental evidence that an increase in synapse dynamics underlies the pathophysiology in mouse models of ASDs.

The introduction of fluorescence imaging in neurobiology revealed that the neuronal cytoskeleton and synaptic structure are not static but dynamic cellular components. Imaging technology is expected to further advance our understanding of the dynamic properties of neurons and neural circuits.

## Introduction

Neural circuits in the cerebral cortex and hippocampus mature progressively during the postnatal period, and their function is adjusted according to sensory information and experiences. The regulated interaction between the sensory input and neural circuits requires the proper expression of functional molecules necessary for circuit construction. Furthermore, intracellular transport of these functional molecules at the right time to the right place is essential. Novel technology that records the morphological and biochemical properties of living neurons is required to study these processes.

**Figure 1.**
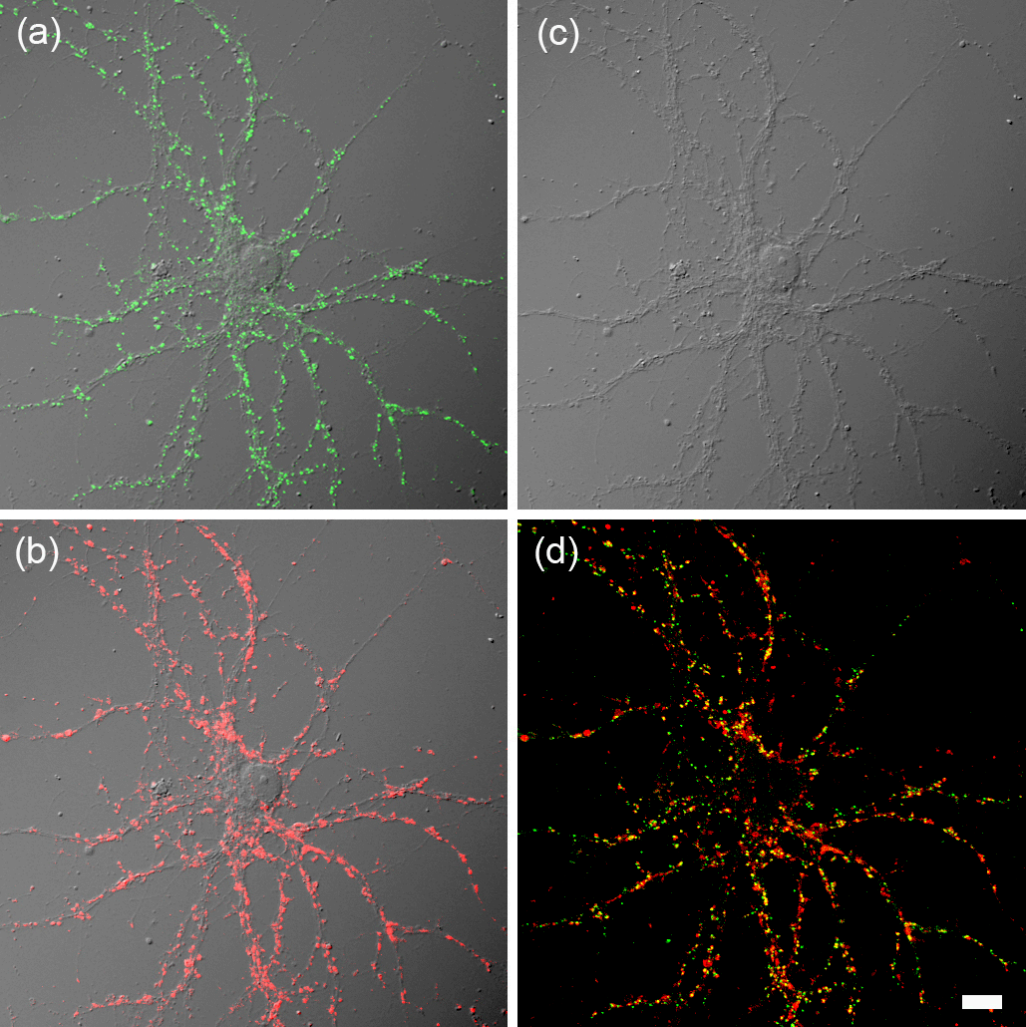
Immunocytochemical labeling of pre- and postsynaptic molecules in cultured hippocampal neurons illustrates the distribution of numerous synaptic connections formed onto branching dendrites. (a) Distribution of the postsynaptic protein, Homer1 (green) overlaid with a differential interference contrast (DIC) image. (b) Distribution of the presynaptic protein, vesicular glutamate transporter 1 (VGLUT1; red) overlaid with a DIC image. (c) DIC image of the same neuron exhibiting the pattern of branching dendrites. (d) Overlaid image of Homer1 (green) and VGLUT1 (red). Bar, 10 μm.

In the field of neuroscience, extensive research on neural circuits in the cerebral cortex and hippocampus has been conducted. The neuron, which is the basic unit of a neural circuit, establishes its polarity and extends intricately branched dendrites and extremely long axons. When the elongating axon comes into contact with a target neuron, synapses, which are special intercellular adhesion structures, are formed (Figure 1). Most synapses in the cerebral cortex and hippocampus are chemical synapses that release neurotransmitters from the presynaptic site (corresponds to the axon terminal) and receive the neurotransmitters via receptors located at the postsynaptic site (corresponds to structures called dendritic spines, protruding from dendritic shafts). Synapse formation begins in the embryonic brain, but the number of synapses increases rapidly in the early postnatal period. After the peak of synaptogenesis during the ages of 3-10 years in the human cerebral cortex, the number of synapses slowly declines.

The manner in which the brain processes and stores information is primarily determined by the specificity of connections through synapses. How connections between neurons through specific synapses are achieved during brain development is one of the most critical questions in neuroscience. To elucidate this, it is necessary to understand the molecular mechanisms by which neurons form specialized patterns of dendrites and axons. It is also essential to clarify the detailed process of synapse formation and maturation.

## Visualization of the Cytoskeletal Dynamics in the Axon

Of the two types of processes formed by neurons, axons, and dendrites, a large amount of research has been conducted on the molecular mechanisms of axon elongation. Axons are more than a meter long in some animals, and surprisingly, a single cell can maintain such an extremely long process over a long period of time. When axons elongate, assembly of the cytoskeleton (three major types: microtubules, actin filaments, and intermediate filaments) is required. Therefore, the mechanisms of cytoskeletal assembly in the axon play a central role in axonal growth. In addition to the cytoskeletal assembly in the axon, the role of growth cones, a unique structure rich in actin cytoskeleton at the tip of the growing axon, is important in understanding the mechanical process of axon elongation.

Until around 1980, research on axon elongation and maintenance relied on two major techniques: the analysis of proteins labeled with radioactive isotopes in the axoplasm and the structural study of chemically fixed axons using electron microscopy. However, these studies had difficulty in answering questions about how proteins are transported and assembled to form polymers. The localization and accumulation of protein molecules are determined by their physicochemical properties and interactions with other molecules. Therefore, to understand the behavior of cytoskeletal proteins within axons, it is necessary to develop a technique to visualize specific molecular species within living cells.

To visualize the dynamics of proteins that form cytoskeletal polymers, a method has been developed in which cytoskeletal proteins such as actin and tubulin are biochemically purified, labeled with fluorescent molecules, and then introduced into nerve cells via microinjection ^(1)^. This technique revealed for the first time the dynamics of the three types of cytoskeleton within neurons. Contrary to the prevailing hypothesis that within axons, the cytoskeletal structure is highly stabilized and transported as interconnected structures, the imaging experiment revealed that the cytoskeleton undergoes continuous cycles of polymerization and depolymerization within the axon. Notably, axon elongation is driven by the addition of new cytoskeletal subunits at the axon tip ^(2), (3)^. The conventional model of “slow axonal transport” was significantly transformed by introducing the perspective that local cycles of polymerization and depolymerization supply the material necessary for axon elongation.

## Visualization of the Synapse Dynamics

Electrophysiological techniques have been used to study synapse formation, which determines the specificity of neural circuits. Although electrophysiology can detect functional synapses, it is not suitable for tracking the formation of many synapses. In cases where neurons with complex dendrites form thousands of synapses, such as those in the mammalian cerebral cortex and hippocampus, electrophysiology is not a suitable method for studying synapse formation. Another promising method for tracking synapse formation is light microscopic imaging. In the 1990s, advances were made in the identification of molecules localized in the presynaptic and postsynaptic sites, which opened up the possibility of using these molecules as imaging probes. Around the same period, GFP, the fluorescent protein, was introduced and proved to be useful as a fusion molecule with other proteins, raising expectations for the application of GFP-labeled synaptic proteins for live cell imaging.

In line with this general expectation in the research field, we explored the possible application of a protein molecule called postsynaptic density protein 95 (PSD-95), which accumulates in postsynaptic structures, to identify synapses by expressing a fusion molecule of PSD-95 and GFP in neurons. In 1999, our group’s first report showed that live imaging of postsynaptic structures was possible ^(4), (5)^. Using this technology, we can monitor the dynamic behavior of synapses formed on dendrites over long periods. These experiments showed that many newly formed synapses during development were eliminated later and that only a subset of new synapses survived and stabilized ^(6)^. Synapse formation and removal occurred simultaneously, and the balance was biased toward formation, resulting in a gradual increase in the overall synapse density during development. It was also shown that after the peak of synapse increase, both formation and removal were suppressed, with synapse removal slightly exceeding formation, resulting in a slow decline in the total number of synapses thereafter. The hypothesis that the balance between formation and deletion is essential in the developmental process of neural circuits was initially proposed from observations of cultured neurons. Subsequently, synapse dynamics in living mice were investigated using two-photon excitation microscopy, and the balanced synapse formation and elimination hypothesis was confirmed.

Using synapse probes such as GFP-labeled PSD-95 led to the progress in elucidating the properties of the protein complex, postsynaptic density (PSD). The molecules that constitute PSD are incorporated into and dissociated from PSD within minutes ^(7)^. This rapid molecular exchange is necessary for the flexible formation and removal of the synapse itself. Furthermore, a method of quantifying the absolute number of molecules using GFP-labeled PSD proteins revealed that the PSD structure within a single synapse contains approximately 300 scaffold proteins, including PSD-95 ^(8)^.

## Imaging the Diversity of Synapse Development

Different brain regions are responsible for specific functions, and local circuits in these regions are interconnected to perform even more complex information processing tasks. Each brain region has a unique local circuit that is evolutionally shaped to execute its specialized function, and the developmental process of synaptic connections determines the uniqueness of the local circuit. Synapse formation mechanisms are thought to be different for each brain region and the type of neuron. Because research on the dynamic process of synapse formation is technically challenging, most studies have been limited to excitatory synapses in the cerebral cortex and hippocampus. The development of synapse imaging technology enabled us to directly monitor the dynamic behavior of heterogeneous types of synapses ^(9), (10), (11)^.

Many neurons in the cerebral cortex and hippocampus are excitatory neurons that release glutamate as a neurotransmitter; however, some inhibitory neurons that use GABA as a transmitter are present. These GABAergic inhibitory neurons control the overall level of excitation in local circuits. Excitatory synapses formed on inhibitory neurons are essential for controlling the excessive excitation of circuits; however, the development of these synapses is poorly understood. Synapse imaging has shown that synapses actively move on long filopodia-like projections formed on the dendrites of inhibitory neurons ^(9)^. This phenomenon indicates that inhibitory neurons can actively search for surrounding axons to find appropriate presynaptic targets. This study also showed that each type of neuron has a unique strategy for synapse formation.

Purkinje cells are large inhibitory neurons in the cerebellum. However, excitatory synapses formed on the dendrites of Purkinje cells do not form long filopodia-like structures. In contrast, axons from granule cells form circular structures that surround Purkinje cell spines and promote synapse maturation ^(10)^. The cerebellum is the control center for smooth body movements and forms local circuits distinct from those of the cerebral cortex and hippocampus. This study also demonstrates the diversity in the mechanisms of synapse formation.

## Imaging Synapse Dynamics in Developmental Disorders

Using imaging technology, we have clarified the dynamic changes in synapses that accompany the development of neural circuits. If such knowledge can be applied to human diseases, it may contribute to the development of new strategies for diagnosis, treatment, and prevention. Human genetics has suggested that autism spectrum disorder (ASD), an early-onset disorder in human brain development, may be related to impairments in synapse development. However, there have been few effective approaches that use animal models to understand the pathology underlying ASDs.

Recently, researchers have established multiple mouse models containing genetic mutations identified in patients with ASD. If we can identify common circuit-level phenotypes in these mice, the translation of knowledge obtained from mouse models to human patients will be promoted. A study that directly measured synapse turnover in the cerebral cortex using two-photon excitation microscopy in three ASD mouse models showed that the synapse turnover rate was approximately doubled in all three models ^(12)^. This result suggests that excessive synapse formation and removal impairs accurate synaptic connections, affecting proper information processing in the cerebral cortex. It is crucial to extract ASD-related circuit-level phenotypes from mouse models because information about circuit impairments in mice could be directly linked to the underlying pathophysiology of the disease.

## Conclusion

The specificity of local neural circuits and their integration are required for higher brain functions, and the core of this circuit specificity is the precise development of synaptic connections. To answer the question of how neuronal connections through specific synapses are achieved during brain development, microscopic imaging of neuron morphogenesis and synapse development is essential. Recent developments in imaging technology are remarkable and significantly contributed to the extraction of information about molecular dynamics and nanoscale structures ^(13), (14)^. Among the new microscope technologies, *in vivo* imaging of large brain regions using high temporal resolution is essential. Another important direction is the development of technologies that can detect the fine structures of synapses with a resolution close to that of electron microscopy. New microscope technologies will advance the field of neural circuit formation and provide exciting new findings that will contribute to our understanding of brain function and related diseases.

## Article Information

This article is based on the study, which received the Medical Award of The Japan Medical Association in 2023. This is a revised English version of the article originally published in Japanese in the Journal of the Japan Medical Association 2023;152(10):1151-4 ^(15)^. The original version is available at https://med.or.jp/cme/jjma/newmag/pdf/152101151.pdf. Only members of the Japan Medical Association are able to access it.

### Conflicts of Interest

None

### Sources of Funding

This work was supported by Grants-in-Aid for Scientific Research (20H05894, 20H05895 to S.O.) and the Japan Agency for Medical Research and Development (JP19gm1310003, JP22jm0210097 to S.O.).

### Acknowledgement

The author thanks all the present and previous members of the Okabe Laboratory for their contributions to the work mentioned in this manuscript.

### Approval by Institutional Review Board (IRB)

The approval code was issued by the institutional review board, and the institution(s) that granted the approval was as follows: Institutional Review Board of The University of Tokyo (approval number 70; June 20, 2023).
